# Symptomatic vs. Asymptomatic 20–40% Internal Carotid Artery Stenosis: Does the Plaque Size Matter?

**DOI:** 10.3389/fneur.2019.00960

**Published:** 2019-10-01

**Authors:** Ahmed Mohamed Elhfnawy, Jens Volkmann, Mira Schliesser, Felix Fluri

**Affiliations:** ^1^Department of Neurology, University Hospital Würzburg, Würzburg, Germany; ^2^Department of Neurology, University Hospital of Essen, Essen, Germany; ^3^Department of Neurology, University Hospital of Alexandria, Alexandria, Egypt; ^4^Department of Neurology, Kantonssptial St. Gallen, St. Gallen, Switzerland

**Keywords:** ischemic stroke, carotid atherosclerosis, carotid stenosis, plaque cross-sectional area, length of stenosis, carotid ultrasound

## Abstract

**Background:** Around 9–15% of ischemic strokes are related to internal carotid artery (ICA)-stenosis ≥50%. However, the extent to which ICA-stenosis <50% causes ischemic cerebrovascular events is uncertain. We examined the relation between plaque cross-sectional area and length and the risk of ischemic stroke or TIA among patients with ICA-stenosis of 20–40%.

**Methods:** We retrospectively identified patients admitted to the Department of Neurology, University Hospital of Würzburg, from January 2011 until September 2016 with ischemic stroke or TIA and concomitant ICA-stenosis of 20–40%, either symptomatic or asymptomatic. Plaque length and cross-sectional area were assessed on ultrasound scans.

**Results:** We identified 41 patients with ischemic stroke or TIA and ICA-stenosis of 20–40%; 14 symptomatic and 27 asymptomatic. The plaque cross-sectional area was significantly larger among symptomatic than asymptomatic ICA-stenosis; median values (IQR) were 0.45 (0.21–0.69) cm^2^ and 0.27 (0.21–0.38) cm^2^, *p* = 0.03, respectively. A plaque cross-sectional area ≥0.36 cm^2^ had a sensitivity of 71% and a specificity of 76% for symptomatic compared with asymptomatic ICA-stenosis. In a sex-adjusted multivariate logistic regression, a plaque cross-sectional area ≥0.36 cm^2^ and a plaque length ≥1.65 cm were associated with an OR (95% CI) of 5.54 (1.2–25.6), *p* = 0.028 and 1.78 (0.36–8.73), *p* = 0.48, respectively, for symptomatic ICA-stenosis.

**Conclusion:** Large plaques might increase the risk of ischemic stroke or TIA among patients with low-grade ICA-stenosis of 20–40%. Sufficiently powered prospective longitudinal cohort studies are needed to definitively test the stroke risk stratification value of carotid plaque length and cross-sectional area in the setting of current optimal medical treatment.

## Introduction

Internal carotid artery (ICA)-stenosis ≥50% causes around 9–15% of ischemic strokes (1). Evidence is accumulating that low-grade ICA-stenosis bears also a high-risk for ischemic stroke (2–5); if no optimal medical treatment is implemented, the annual ipsilateral stroke rate associated with mild-to-moderate asymptomatic ICA-stenosis is 0.1–1.6% compared to 2–3.3% among severe stenotic degrees (6, 7). Previous studies pointed to carotid plaque area as a useful parameter to stratify the risk of stroke and even to monitor preventive treatment of cerebrovascular and major vascular diseases (8, 9). In the current work, we investigated whether the carotid plaque cross-sectional area and/or length are more prominent among symptomatic than asymptomatic ICA-stenosis of 20–40%.

## Methods

### Study Design

We conducted a subgroup analysis from a recently published cohort (10). In the current retrospective observational study, we included patients with an acute ischemic stroke or TIA admitted within 14 days of symptom onset, and a concomitant ipsilateral symptomatic ICA-stenosis of 20–40% (measured according to the NASCET criteria) without any other apparent stroke etiology according to the Trial of Org 10,172 in Acute Stroke Treatment (TOAST) (11), or a contralateral asymptomatic ICA-stenosis of 20–40% in the Department of Neurology (University Hospital of Würzburg) from January 2011 until September 2016. The exclusion criteria were: (1) ischemic stroke due to coronary angiography, carotid artery stenting, or carotid endarterectomy (CEA), (2) absence of ultrasound scans, or (3) bilateral stroke. In patients with bilateral ICA-stenosis of 20–40%, we included the symptomatic side in the present study and excluded the asymptomatic one to avoid including the same patient in the two study groups. All patients received the following minimal stroke work-up: routine blood tests within 24 h after index-TIA or index-stroke, ECG-monitoring with atrial fibrillation (AF) alarm for a minimum of 24-h, transthoracic echocardiography, extra- and transcranial color-coded duplex ultrasonography, and neuroimaging using computed tomography (CT) and/or magnetic resonance imaging (MRI).

Stroke was defined as acute-onset focal neurological deficits with a corresponding brain lesion in a vascular distribution showing hyperintensity in the diffusion-weighted images (DWI) with a corresponding hypointensity in the apparent diffusion coefficient (ADC) images and/or hypodensity in the cerebral CT. If the patient did not undergo cerebral MRI, stroke was diagnosed if the deficits persisted >24 h, with no other reasonable etiology. TIA was diagnosed if the deficits did not lead to brain lesions in the neuroimaging and the deficits persisted <24 h. Hypertension was defined as the presence of any of the followings: (1) persistent elevation of the systolic blood pressure ≥140 mmHg or diastolic blood pressure ≥90 mmHg requiring the initiation of antihypertensive drugs (12), or (2) if the patient was previously diagnosed with hypertension and has already received antihypertensive medications. Diabetes mellitus was defined according to the following criteria: (1) Hemoglobin A1c ≥ 6.5%, (2) Fasting blood glucose ≥126 mg/dl, (3) Blood glucose ≥200 mg/d, 2 h after oral glucose tolerance test (13), or (4) Previous diagnosis and treatment of diabetes mellitus. The patients were diagnosed as active smokers if they regularly consumed cigarettes within the previous 12 months. Atrial fibrillation (AF) was diagnosed according to the following criteria: (1) previously documented AF in the family physician's office prior to the index event or (2) 12-lead ECG or Holter ECG recorded after the index event showing loss of *p*-waves with irregular rapid heart rate and narrow QRS-complex for a duration of at least 30 s.

### Ultrasound Assessment of the Plaque Length and Cross-Sectional Area

**Figure 1:**
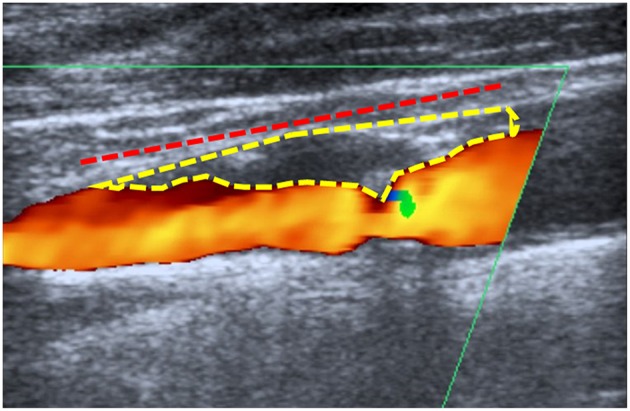
Measurement method: Example for the method used to measure the plaque length (dashed red line) and cross-sectional area (dashed yellow) in a patient with symptomatic low-grade internal carotid artery stenosis of 20–40%.

Ultrasound examination was conducted on a Toshiba AplioXG machine (Toshiba Medical Systems Corporation, Tochigi, Japan) using a 7.5 MHz linear array transducer. Ultrasound images were assessed in our picture archiving and communication system (PACS) by a single non-blinded examiner (AE). The hemodynamic criteria of the NASCET were used to measure the degree of stenosis (14). The stenotic length was measured from the most proximal to the most distal stenotic end, in the projection demonstrating the longest length, using a previously published method (10). The stenotic ends were defined according to the following criteria: (1) visible narrowing of the vascular lumen, (2) flow turbulence in the proximal stenotic end. For the distal end, this criterion was used to identify the distal end only in the presence of Doppler waves showing increased flow velocity. This is because there is no possible differentiation between a flow turbulence resulting from increased systolic flow velocity and that resulting from poststenotic flow disturbance, and/or (3) calcification shadowing. For the assessment of the plaque cross-sectional area, we used a previously published method (8, 15). First, we identified the projection demonstrating the largest cross-sectional area. Then we magnified the stored images in our PACS. Thereafter, we outlined the plaque edges using a cursor. Finally, the plaque cross-sectional area was displayed on the screen. Figure 1 demonstrates the measurement methods used for the plaque length and cross-sectional area.

### Statistical Analysis

Quantitative data were expressed using median and interquartile range, while qualitative data were expressed in absolute values and relative frequencies. To check for normality, we used a histogram and the Shapiro-Wilk test. Univariable statistical tests were conducted using χ2 test or Fischer exact test (if *n* < 5) for categorical data as well as Mann-Whitney *U*-test for continuous data. We calculated the sensitivity and specificity of plaque length and cross-sectional area using a receiver operating characteristic (ROC)-curve. An AUC > 0.5 indicates better prediction; values closer to 1 indicate more accurate prediction. Univariate binary logistic regression analyses were performed to measure the strength of association, measured as OR (95% CI), between the occurrence of ischemic stroke or TIA in relation to low-grade ICA-stenosis and other possibly related variables. Variables with a *p* < 0.1 and our two study variables (plaque cross-sectional area as well as plaque length) were further assessed in a multivariate logistic regression model. The fitness of this model was tested using a Hosmer-Lemeshow “goodness-of-fit” test. Data were analyzed in SPSS software package version 25 (SPSS, Chicago, IL, USA). *P* < 0.05 was considered statistically significant.

## Results

**Figure 2:**
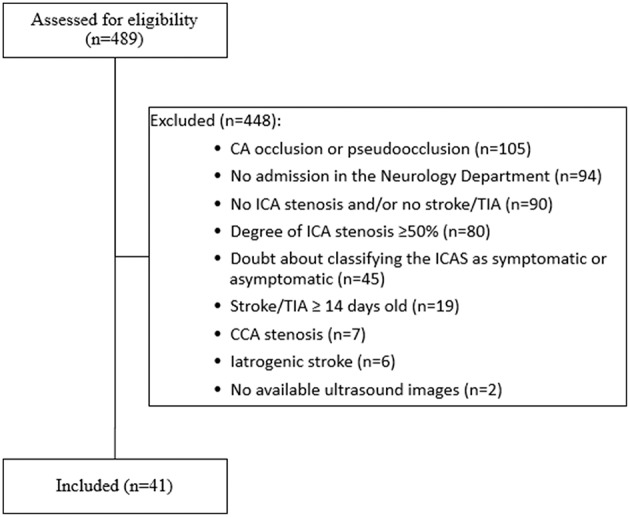
Flow chart showing the inclusion and exclusion criteria for patients enrolled in the present study. CA, carotid artery; CCA, common carotid artery; ICA, internal carotid artery; TIA, transient ischemic attack, iatrogenic stroke (5 cases after carotid endarterectomy, 1 case after coronary angiography). Of the 489 patients screened, only 41 patients met our inclusion and exclusion criteria.

**Figure 3:**
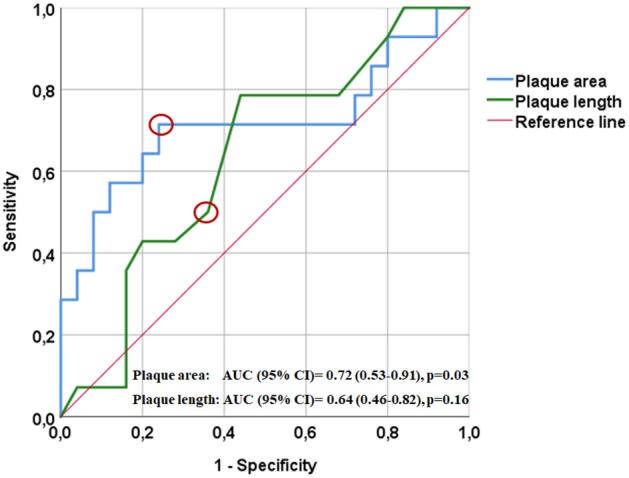
Receiver operating characteristics curve (ROC) showing the relation between plaque surface area (blue) and length (green) with ipsilateral ischemic manifestations. A cut-off plaque cross-sectional area of ≥0.36 cm^2^ (indicated by the red circle) yielded a sensitivity of 71% and a specificity of 76% for the development of ipsilateral manifestations; AUC (95% CI) = 0.72 (0.53–0.91), *p* = 0.03. A cut-off plaque length of ≥1.65 cm (indicated by the red circle) yielded a sensitivity of 50% and a specificity of 64% for the development of ipsilateral manifestations; AUC (95% CI) = 0.64 (0.46–0.82), *p* = 0.16.

Four hundred eighty-nine patients, who received the ICD10-codes for stroke or TIA as well as ICA-stenosis, were screened for our inclusion and exclusion criteria. Forty-one patients (27 asymptomatic and 14 symptomatic ICA-stenosis) were included (See flow chart, Figure 2). The baseline characteristics are shown in Table 1. The risk factors were similar between the two groups. The plaque cross-sectional area was significantly larger and the length was insignificantly longer for symptomatic than asymptomatic ICA-stenosis; median (IQR) were 0.45 (0.21–0.69) cm^2^ and 0.27 (0.21–0.38) cm^2^, *p* = 0.03, as well as 1.7 (1.5–2.1), and 1.4 (1.1–1.8) cm, *p* = 0.15, respectively. Using a ROC-curve (Figure 3), a plaque cross-sectional area ≥0.36 cm^2^ and a plaque length ≥1.65 cm were found to have a sensitivity of 71 and 50% and a specificity of 76 and 64%, respectively, for symptomatic compared to asymptomatic ICA-stenosis (for cross-sectional area: AUC 0.72, 95% CI 0.53–0.91, *p* = 0.03, whereas for length: AUC 0.64, 95% CI 0.46–0.82, *p* = 0.16). In a sex-adjusted multivariate binary logistic regression model (Table 2), a plaque cross-sectional area ≥0.36 cm^2^ and a plaque length ≥1.65 cm were associated with an OR (95% CI) of 5.54 (1.2–25.6), *p* = 0.028, and 1.78 (0.36–8.73), *p* = 0.48, respectively, for symptomatic compared to asymptomatic ICA-stenosis. The Hosmer-Lemeshow “goodness-of-fit” test showed a non-significant difference between the observed and expected results with *p* = 0.076.

**Table 1:** Baseline characteristics.

**Characteristics**	**Asymptomatic ICA-stenosis (*n* = 27)**	**Symptomatic ICA-stenosis (*n* = 14)**	***P*-value**
Age-year, median (IQR)	74 (67–81)	75 (59–83)	0.86
Male sex, no. (%)	13 (48.1)	11 (78.6)	0.1
Hypertension, no. (%)	26 (96.3)	12 (85.7)	0.27
Diabetes mellitus, no. (%)	10 (37)	4 (28.6)	0.73
Active smoking, no. (%)	6 (22.2)	3 (21.4)	1
AF, no. (%)	11 (40.7)	Exclusion criteria	
Previous treatment			
Antiplatelets, no. (%)	14 (51.9)	9 (64.3)	
Anticoagulants, no. (%)	6 (22.2)	2 (14.3)	
Statins, no. (%)	5 (18.5)	7 (50)	
Antihypertensive drugs, no. (%)	19 (70.4)	11 (78.6)	
Ischemic cerebrovascular event			0.72
Stroke, no. (%)	20 (74.1)	9 (64.3)	
TIA, no. (%)	7 (25.9)	5 (35.7)	
Time from stroke onset to admission-days, median (IQR)	1 (1–1)	1 (1–2)	
NIHSS-score on admission, median (IQR)	2 (0–7)	1 (0–3)	0.22
HbA1c (%), median (IQR)	6.1 (5.7–7.2)	6.2 (5.4–6.5)	0.45
LDL-cholesterol (mg/dl), median (IQR)	115 (87.5–164)	105 (94–133)	0.57
Hemoglobin (mg/dl), median (IQR)	13.6 (12–14.7)	14.1 (13.7–14.8)	0.16
Plaque size in cm^2^, median (IQR)	0.27 (0.21–0.38)	0.45 (0.21–0.69)	0.03^*^
Plaque size ≥ 0.36 cm^2^, no. (%)	7 (25.9)	10 (71.4)	0.008^*^
Plaque length in cm, median (IQR)	1.4 (1.1–1.8)	1.7 (1.5–2.1)	0.15
Acute treatment with IV alteplase, no. (%)	4 (14.8)	2 (14.3)	1

* *Statistically significant results*.

*AF, atrial fibrillation; HbA1c, hemoglobin A1c; ICA, internal carotid artery; IQR, interquartile range; LDL-cholesterol, low density lipoprotein cholesterol; mRS, modified Rankin scale; NIHSS, national institute of health stroke scale; TIA, transient ischemic attack*.

**Table 2:** Factors related to the occurrence of stroke or transient ischemic attack ipsilateral to a low-grade stenosis in the binary logistic regression models.

**Characteristic**	**Univariate regression analysis**			**Multivariate regression analysis**		
	***OR***	**95% CI**	***P***	***OR***	**95% CI**	***P***
Age	0.98	0.93–1.05	0.6			
Male sex	3.91	0.9–17.4	0.07	3.7	0.7–19.45	0.12
Hypertension	0.23	0.02–2.8	0.25			
Diabetes mellitus	0.68	0.17–2.75	0.59			
Active smoking	0.96	0.2–4.57	0.95			
HbA1c (%)	0.77	0.39–1.54	0.46			
LDL-cholesterol (mg/dl)	0.99	0.98–1.01	0.35			
Hemoglobin (mg/dl)	1.56	0.91–2.67	0.1			
Plaque length ≥1.65 cm	2	0.54–7.47	0.3	1.78	0.36–8.73	0.48
Plaque cross–sectional area ≥0.36 cm^2^	7.14	1.69–30.27	0.008^*^	5.54	1.2–25.6	0.028^*^
H-L-Test^a^						0.076

* *Statistically significant results*.

*CI, confidence interval; HbA1c, hemoglobin A1c; LDL-cholesterol, low-density lipoprotein cholesterol; OR, odds ratio*.

a *Hosmer-Lemeshow “goodness-of-fit test for the multivariate regression analysis showed a non-significant p-value (p = 0.076) for the difference between our observed results and the expected results. The non-significant p-value for this test means better fit of the model (the higher the value, the better the fit)*.

## Discussion

### Plaque Size May Represent a High-Risk Plaque Feature

We found that the plaques of symptomatic low-grade ICA-stenosis of 20–40% had a significantly larger cross-sectional area and were insignificantly longer in comparison to asymptomatic ICA-stenosis of 20–40%. Among our patients with ICA-stenosis of 20–40%, a plaque cross-sectional area ≥0.36 cm^2^ had a sensitivity of 71% and a specificity of 76% with an OR (95% CI) of 5.54 (1.2–25.6) for symptomatic ICA-stenosis. We measured the plaque area using a method similar to previous studies. Taking the paucity of studies on plaque length into account, we considered several elements (narrowing of the vascular lumen, flow turbulence, increased flow velocity, and plaque calcification) to increase the accuracy of our measurement method for the plaque length. In line with our findings, the asymptomatic carotid stenosis and risk of stroke study (ACSRS) showed that increased plaque area, measured on ultrasound, is associated with increased future ipsilateral stroke rate from asymptomatic ICA-stenosis of 12–99% (NASCET) (15). The median (IQR) plaque area in the ACSRS study was 0.42 (0.27–0.6) cm^2^ and plaques >0.8 cm^2^ were noticeably associated with higher ipsilateral stroke risk. Of note, the ACSRC study included ICA-stenosis of 12–99%, whereas our study was restricted to ICA-stenosis of 20–40%. Moreover, previous studies reported that plaque thickness, length, and volume were more prominent on the ipsilateral side among patients with stroke (2, 4). Similarly, another study showed that carotid plaque volume of the endarterectomy specimens was larger in symptomatic than in asymptomatic ICA-stenosis (mean, 0.97 vs. 0.74 cm^3^, *p* < 0.001) (16). Furthermore, the total areas of all plaques in the right carotid artery, measured on ultrasound, were a strong predictor of ischemic stroke (9). Another study showed that the total carotid plaque areas on ultrasound in both carotid arteries were positively related to the combined 5-year risk of stroke, myocardial infarction, and vascular death (8). In a population-based study, larger plaque thickness (17), or carotid intima-media thickness (18, 19) were found to bear a higher risk for future major vascular events. Conversely, Nandalur et al. reported no difference between carotid plaque volume, measured by multidetector CT, among symptomatic compared to asymptomatic ICA-stenosis (20). Of note, a prominent inverse correlation between the degree and length of stenosis was reported among patients with symptomatic ICA-stenosis ≥ 70% but neither among symptomatic ICA-stenosis <70% nor among asymptomatic ICA-stenosis (10). Interestingly, statin was found to slow plaque progression, and even led to regression of the carotid plaques. This effect was mainly shown with maintaining the serum level of low-density lipoprotein <100 mg/dl independent of the statin dose (21). Plaque size can be used to stratify the cardio- or cerebrovascular risk and to monitor response to treatment (8, 15).

### Other High-Risk Plaque Features

Several researchers investigated high-risk plaque features. Demarco et al. found that the following plaque features, assessed on magnetic resonance angiography (MRA), were more frequent among symptomatic mild-to-moderate ICA-stenosis compared to asymptomatic mild-to-moderate stenosis: thin or ruptured fibrous cap (100 vs. 36%, *p* = 0.006), lipid-rich necrotic core (100 vs. 39%, *p* = 0.022), and -to a lesser extent- plaque hemorrhage (86 vs. 33%, *p* = 0.055) (5). A meta-analysis showed that intraplaque hemorrhage (IPH), defined as the presence of a hyperintense signal within the carotid plaque on T1-weighted fat-suppressed MRI images, is associated with an annual stroke/TIA rate of 17.71% compared to 2.43% in relation to plaques without hemorrhage (22). The authors noted that IPH was associated with a hazard ratio [HR] of 5.69 (95% CI 2.98–10.87) for stroke/TIA. However, optimal medical treatment was not accurately implemented for patients included in the aforementioned meta-analysis; only 62% of the patients had a statin and 72% had an antiplatelet therapy, whereas data regarding further elements of optimal medical treatment are not available. Another meta-analysis identified the following high-risk ultrasound plaque features: plaque neovascularity (OR 19.68, 95% CI 3.14–123.16), complex plaque (OR 5.12, 95% CI 3.42–7.67), plaque ulceration (OR 3.58, 95% CI 1.66–7.71), plaque echolucency (OR 3.99, 95% CI 3.06–5.19), and intraplaque motion (OR = 1.57, 95% CI = 1.02–2.41) (23). Of note, hypoechoic plaque morphology is more often associated with intraplaque hemorrhage (24), and is notably related to fatal or disabling stroke (15). Plaque inflammation on PET/CT was also identified as a high-risk feature (25). Interestingly, the ACSRS study showed that the combination of high-risk clinical features, high-risk plaque features, and the degree of stenosis could better stratify the risk of ipsilateral stroke/TIA (15). The authors of the aforementioned study identified the following high-risk plaque features: hypoechoic plaque morphology or low gray scale median (GSM), increased plaque area, and the presence of discrete white areas without acoustic shadowing. For example, the authors showed a 5-year ipsilateral stroke rate of 70.4% among patients with the following combinations: asymptomatic ICA-stenosis of 50–82% (NASCET), history of contralateral stroke/TIA, discrete white areas, plaque area >0.8 cm^2^, and low GSM <15. Conversely, the 5-year ipsilateral stroke rate was as low as 1.9% for patients with the following combinations: asymptomatic ICA-stenosis 83–99% (NASCET), no history of contralateral stroke/TIA, absence of discrete white areas, plaque area <0.4 cm^2^, and GSM >30. Of note, the ACSRS study was conducted before the era of optimal medical treatment, so that the aforementioned 5-year ipsilateral stroke rates of 70.4% and 1.9% should be viewed with caution. This elucidates the importance of risk stratification using multiple high-risk clinical and plaque features rather than a single one. The follow-up of these features might be used to optimize medical treatment or to investigate new medical therapies for cerebrovascular and other major vascular events.

### High-Risk Plaque Features Represent a Fruitful Subject for Future Research

More than 20 years ago, CEA was shown to provide an overall stroke prevention benefit in symptomatic patients with ICA-stenosis ≥50% (26–29). However, these data should be interpreted with caution, because they were conducted before the introduction of optimized medical treatment. The latter includes antiplatelets, statins, control of blood pressure and blood glucose level, smoking cessation, implementation of exercise programs, Mediterranean diet, and lifestyle modification, and was shown to significantly reduce the stroke risk among patients with asymptomatic ICA-stenosis (30, 31). Current optimized medical treatment is an established therapy for prevention of arterial disease complications, including patients with carotid stenosis, whether mild or severe, and it is a continually improving gold standard of care (32, 33), and should also be offered perioperatively before CEA (34). Best medical treatment seems to be as effective as CEA or carotid artery stenting for asymptomatic moderate-to-severe ICA-stenosis (30, 31, 33). Additionally, medical treatment is around 3–8 times more cost-effective than carotid procedures (33). Of note, under suboptimal medical treatment, the annual ipsilateral stroke rate associated with mild-to-moderate asymptomatic ICA-stenosis is 0.1–1.6% and 2–3.3% in severe asymptomatic ICA-stenosis (6, 7). Under best medical treatment, an average annual stroke rate among moderate-to-severe asymptomatic ICA-stenosis ranges from 0.3–3.1% (31). In the current work, large-sized plaques were shown to represent a high-risk plaque feature. The incidence of ipsilateral stroke associated with high-risk plaque features, and the protective effect of best medical treatment, as well as carotid procedures for those high-risk plaques, remain under-investigated. We recommend the assessment of this risk in future observational studies. If any subgroup can be identified with high-risk for ipsilateral stroke, despite best medical treatment, it is important to further test the cost-benefit ratio of carotid procedures for those patients in randomized trials before the results can be translated into the clinical practice.

### Study Limitations

We are aware of the following limitations: First, the sample size is small. However, this study was planned as a pilot study and serves as a proof-of-concept. Second, the design of the present study is retrospective and has to be confirmed in large prospective observational studies to stratify the risk related to different degrees of ICA-stenosis and high-risk plaque features. This allows assessing whether there is a subgroup with an ipsilateral stroke rate high enough to justify the conduction of trials of more intensive/different medical intervention and/or carotid procedures. Third, the assessment of the plaque length and surface area was performed by a non-blinded investigator, which represents another study limitation. Fourth, there is a bias of a sub-group analysis from a non-predefined primary outcome. Fifth, other individual risk factors, other plaque characteristics, and previous medications may have influenced our comparative analysis.

## Conclusion

Large plaques might represent a high-risk marker for further complication of arterial disease including ipsilateral stroke among patients with low-grade ICA-stenosis. Sufficiently powered prospective longitudinal cohort studies are needed to definitively test the stroke risk stratification value of carotid plaque length and cross-sectional area in the setting of current optimal medical treatment. Plaque size, along with other high-risk plaque features and high-risk clinical features should be ideally used to optimize the medical treatment of patients with carotid stenosis as well as other atherosclerotic vascular diseases. Future observational studies are needed to stratify the risk of ipsilateral stroke associated with high-risk clinical features, along with high-risk plaque features among different stenotic degrees. This probably allows to identify subgroups of patients with a risk high enough to justify the conduction of future randomized trials.

## Data Availability

The datasets generated for this study are available on request to the corresponding author.

## Ethics Statement

Data collected within routine clinical care were used. Therefore, no specific approval was needed according to local regulations confirmed by the Ethics Board of the Medical Faculty of the University of Würzburg. Our Ethical Committee was consulted before the conduction of the study and the need for informed consent was waived because of the retrospective nature of the study.

## Author Contributions

AE collected the data, performed the measurements and the statistical analysis, and wrote the first draft. MS contributed to the ultrasound examinations. JV, MS, and FF supervised the work, provided consultations, and revised the manuscript. All authors were involved in the final approval of the version to be published, made a substantial contribution to the conception, design, and revision of the draft.

### Conflict of Interest Statement

The authors declare that the research was conducted in the absence of any commercial or financial relationships that could be construed as a potential conflict of interest.

## References

[1] Abbott AL Bladin CF Levi CR Chambers BR. What should we do with asymptomatic carotid stenosis? Int J Stroke. (2007) 2:27–39. 10.1111/j.1747-4949.2007.00096.x 18705984

[2] Coutinho JM Derkatch S Potvin AR Tomlinson G Kiehl TR Silver FL Non-stenotic carotid plaque on ct angiography in patients with cryptogenic stroke. Neurology. (2016) 87:665–72. 10.1212/WNL.0000000000002978 27412144 PMC4999163

[3] Yamada K Yoshimura S Shirakawa M Uchida K Maruyama F Nakahara S. High intensity signal in the plaque on routine 3d-tof mra is associated with ischemic stroke in the patients with low-grade carotid stenosis. J Neurol Sci. (2018) 385:164–7. 10.1016/j.jns.2017.12.023 29406899

[4] Buon R Guidolin B Jaffre A Lafuma M Barbieux M Nasr N Carotid ultrasound for assessment of non-obstructive carotid atherosclerosis in young adults with cryptogenic stroke. J Stroke Cerebrovasc Dis. (2018) 27:1212–6. 10.1016/j.jstrokecerebrovasdis.2017.11.043 29307510

[5] Demarco JK Ota H Underhill HR Zhu DC Reeves MJ Potchen MJ. Mr carotid plaque imaging and contrast-enhanced MR angiography identifies lesions associated with recent ipsilateral thromboembolic symptoms: an *in vivo* study at 3t. AJNR Am J Neuroradiol. (2010) 31:1395–402. 10.3174/ajnr.A2213 20651015 PMC5659265

[6] Inzitari D Eliasziw M Gates P Sharpe BL Chan RK Meldrum HE. The causes and risk of stroke in patients with asymptomatic internal-carotid-artery stenosis. North American symptomatic carotid endarterectomy trial collaborators. N Engl J Med. (2000) 342:1693–700. 10.1056/NEJM200006083422302 10841871

[7] Nicolaides AN Kakkos SK Griffin M Sabetai M Dhanjil S Tegos T. Severity of asymptomatic carotid stenosis and risk of ipsilateral hemispheric ischaemic events: results from the acsrs study. Eur J Vasc Endovasc Surg. (2005) 30:275–84. 10.1016/j.ejvs.2005.04.031 16130207

[8] Spence JD Eliasziw M DiCicco M Hackam DG Galil R Lohmann T. Carotid plaque area: a tool for targeting and evaluating vascular preventive therapy. Stroke. (2002) 33:2916–22. 10.1161/01.STR.0000042207.16156.B9 12468791

[9] Mathiesen Ellisiv B Johnsen Stein H Wilsgaard T Bønaa Kaare H Løchen M-L Njølstad I. Carotid plaque area and intima-media thickness in prediction of first-ever ischemic stroke. Stroke. (2011) 42:972–8. 10.1161/STROKEAHA.110.589754 21311059

[10] Elhfnawy AM Heuschmann PU Pham M Volkmann J Fluri F. Stenosis length and degree interact with the risk of cerebrovascular events related to internal carotid artery stenosis. Front Neurol. (2019) 10:317. 10.3389/fneur.2019.00317 31024420 PMC6465418

[11] Adams HP Bendixen BH Kappelle LJ Biller J Love BB Gordon DL. Classification of subtype of acute ischemic stroke. Definitions for use in a multicenter clinical trial. Toast. Trial of org 10172 in acute stroke treatment. Stroke. (1993) 24:35–41. 10.1161/01.STR.24.1.35 7678184

[12] Meschia JF Bushnell C Boden-Albala B Braun LT Bravata DM Chaturvedi S. Guidelines for the primary prevention of stroke: a statement for healthcare professionals from the american heart association/american stroke association. Stroke. (2014) 45:3754–832. 10.1161/STR.0000000000000046 25355838 PMC5020564

[13] American diabetes association Diagnosis and classification of diabetes mellitus. Diabetes Care. (2014) 37:S81–90. 10.2337/dc14-S081 24357215

[14] Arning C Widder B von Reutern GM Stiegler H Gortler M. Revision of degum ultrasound criteria for grading internal carotid artery stenoses and transfer to nascet measurement. Ultraschall Med. (2010) 31:251–7. 10.1055/s-0029-1245336 20414854

[15] Nicolaides AN Kakkos SK Kyriacou E Griffin M Sabetai M Thomas DJ. Asymptomatic internal carotid artery stenosis and cerebrovascular risk stratification. J Vasc Surg. (2010) 52:1486–96.e1481–5. 10.1016/j.jvs.2010.07.021 21146746

[16] Ball S Rogers S Kanesalingam K Taylor R Katsogridakis E McCollum C. Carotid plaque volume in patients undergoing carotid endarterectomy. Br J Surg. (2018) 105:262–9. 10.1002/bjs.10670 29315509 PMC5873399

[17] Rundek T Arif H Boden-Albala B Elkind MS Paik MC Sacco RL. Carotid plaque, a subclinical precursor of vascular events. The Northern Manhattan Study. Neurology. (2008) 70:1200–7. 10.1212/01.wnl.0000303969.63165.34 18354078 PMC2831775

[18] O'Leary DH Polak JF Kronmal RA Manolio TA Burke GL Wolfson SK. Carotid-artery intima and media thickness as a risk factor for myocardial infarction and stroke in older adults. N Engl J Med. (1999) 340:14–22. 10.1056/NEJM199901073400103 9878640

[19] Bots Michiel L Hoes Arno W Koudstaal Peter J Hofman A Grobbee Diederick E Common carotid intima-media thickness and risk of stroke and myocardial infarction. Circulation. (1997) 96:1432–7. 10.1161/01.CIR.96.5.1432 9315528

[20] Nandalur KR Hardie AD Raghavan P Schipper MJ Baskurt E Kramer CM. Composition of the stable carotid plaque: insights from a multidetector computed tomography study of plaque volume. Stroke. (2007) 38:935–40. 10.1161/01.STR.0000257995.74834.92 17272781 PMC2966495

[21] Makris GC Lavida A Nicolaides AN Geroulakos G. The effect of statins on carotid plaque morphology: a ldl-associated action or one more pleiotropic effect of statins? Atherosclerosis. (2010) 213:8–20. 10.1016/j.atherosclerosis.2010.04.032 20494361

[22] Saam T Hetterich H Hoffmann V Yuan C Dichgans M Poppert H. Meta-analysis and systematic review of the predictive value of carotid plaque hemorrhage on cerebrovascular events by magnetic resonance imaging. J Am Coll Cardiol. (2013) 62:1081–91. 10.1016/j.jacc.2013.06.015 23850912

[23] Brinjikji W Rabinstein AA Lanzino G Murad MH Williamson EE DeMarco JK. Ultrasound characteristics of symptomatic carotid plaques: a systematic review and meta-analysis. Cerebrovasc Dis. (2015) 40:165–74. 10.1159/000437339 26279159

[24] Tegos TJ Sohail M Sabetai MM Robless P Akbar N Pare G. Echomorphologic and histopathologic characteristics of unstable carotid plaques. AJNR Am J Neuroradiol. (2000) 21:1937–44. 11110550 PMC7974273

[25] Paraskevas KI Spence JD Veith FJ Nicolaides AN. Identifying which patients with asymptomatic carotid stenosis could benefit from intervention. Stroke. (2014) 45:3720–4. 10.1161/STROKEAHA.114.006912 25358698

[26] Rothwell PM Eliasziw M Gutnikov SA Fox AJ Taylor DW Mayberg MR. Analysis of pooled data from the randomised controlled trials of endarterectomy for symptomatic carotid stenosis. Lancet. (2003) 361:107–16. 10.1016/S0140-6736(03)12228-3 12531577

[27] Abbott AL Paraskevas KI Kakkos SK Golledge J Eckstein HH Diaz-Sandoval LJ. Systematic review of guidelines for the management of asymptomatic and symptomatic carotid stenosis. Stroke. (2015) 46:3288–301. 10.1161/STROKEAHA.115.003390 26451020

[28] Barnett HJM Taylor DW Eliasziw M Fox AJ Ferguson GG Haynes RB Benefit of carotid endarterectomy in patients with symptomatic moderate or severe stenosis. N Engl J Med. (1998) 339:1415–25. 10.1056/NEJM199811123392002 9811916

[29] European carotid surgery trialists' collaborative group Randomised trial of endarterectomy for recently symptomatic carotid stenosis: final results of the mrc european carotid surgery trial (ecst). Lancet. (1998) 351:1379–87. 10.1016/S0140-6736(97)09292-1 9593407

[30] Spence JD Song H Cheng G. Appropriate management of asymptomatic carotid stenosis. Stroke Vasc Neurol. (2016) 1:64–71. 10.1136/svn-2016-000016 28959466 PMC5435189

[31] Abbott AL Silvestrini M Topakian R Golledge J Brunser AM de Borst GJ. Optimizing the definitions of stroke, transient ischemic attack, and infarction for research and application in clinical practice. Front Neurol. (2017) 8:537. 10.3389/fneur.2017.00537 29104559 PMC5654955

[32] Loftus IM Paraskevas KI Johal A Waton S Heikkila K Naylor AR. Editor's choice - delays to surgery and procedural risks following carotid endarterectomy in the uk national vascular registry. Eur J Vasc Endovasc Surg. (2016) 52:438–43. 10.1016/j.ejvs.2016.05.031 27364857

[33] Abbott AL Medical (non-surgical) intervention alone is now best for prevention of stroke associated with asymptomatic severe carotid stenosis: results of a systematic review and analysis. Stroke. (2009) 40:e573–83. 10.1161/STROKEAHA.109.556068 19696421

[34] Naylor AR. Optimal medical therapy during carotid endarterectomy: a personal view. Acta Chir Belg. (2009) 109:285–91. 10.1080/00015458.2009.11680427 19943581

